# Supplementary material for: Barriers and facilitators for the use of telehealth by healthcare providers in India—A systematic review
Source: PLOS Digit Health. 2024 Dec 6;3(12):e0000398. doi: 10.1371/journal.pdig.0000398 (PMC11623477; doi:10.1371/journal.pdig.0000398)
Supplement: S2 Panel — (DOCX) [file pdig.0000398.s004.docx]

**S2 Panel:  HR Cadres**

| All doctors and nurses (all doctors plus nurses and midwives) |
| --- |
| All doctors (allopathic plus AYUSH doctors) |
| Ayurvedic, homeopathic and unani (AYUSH) doctors |
| Dental Specialists |
| All Health Professionals |
| Nursing Professionals |
| Medical Assistants |
| Sanitarians |
| Dieticians and Nutritionists |
| Optometrists and Opticians |
| Dental Assistants and Therapists |
| Physiotherapists and Related Associate Professionals |
| Pharmaceutical Assistants |
| Modern Health Associate Professionals (except Nursing) |
| Nursing Associate Professionals |
| Midwifery Associate Professionals |
| Traditional Medicine Practitioners |
| Faith Healers |
