# Supplementary material for: Barriers and facilitators for the use of telehealth by healthcare providers in India—A systematic review
Source: PLOS Digit Health. 2024 Dec 6;3(12):e0000398. doi: 10.1371/journal.pdig.0000398 (PMC11623477; doi:10.1371/journal.pdig.0000398)
Supplement: S2 Table — (DOCX) [file pdig.0000398.s006.docx]

**S2 Table: Risk of bias assessment of included studies**

**S2-a Table: Quality assessment results of quantitative cross-sectional studies using JBI checklist**

| **Study identification** | **Q1** | **Q2** | **Q3** | **Q4** | **Q5** | **Q6** | **Q7** | **Q8** | **Q9** | **% Score** | **Risk of Bias** |
| --- | --- | --- | --- | --- | --- | --- | --- | --- | --- | --- | --- |
| Lindquist et al (73) | Y | N | N | Y | Y | Y | Y | N | Y | 66.7 | Moderate |
| Balakrishnan et al (63) | Y | NA | NA | Y | Y | Y | Y | Y | Y | 77.8 | Low |
| Bairapareddy et al (26) | Y | Y | Y | Y | Y | Y | Y | Y | Y | 100 | Low |
| Usmanova et al (33) | Y | Y | Y | Y | Y | Y | Y | Y | Y | 100 | Low |
| Swathi et al (35) | Y | Y | Y | Y | Y | Y | Y | Y | Y | 100 | Low |
| Ward et al (36) | Y | Y | Y | Y | Y | Y | Y | Y | Y | 100 | Low |
| Verma et al (87) | Y | Y | Y | Y | Y | Y | Y | Y | Y | 100 | Low |
| Nair et al (96) | Y | N | N | Y | Y | Y | Y | Y | Y | 77.8 | Low |
| Naik et al (95) | Y | Y | Y | Y | Y | Y | Y | Y | Y | 100 | Low |
| Ragesh et al (86) | Y | Y | N | Y | Y | Y | Y | Y | Y | 88.9 | Low |
| Acharya et al (83) | Y | N | N | Y | Y | Y | Y | Y | Y | 77.8 | Low |
| Zayapragassarazan et al (72) | Y | Y | Y | Y | Y | Y | Y | Y | Y | 100 | Low |
| Patterson et al (58) | Y | N | N | Y | Y | Y | Y | Y | Y | 77.8 | Low |
| Garner et al (59) | Y | N | N | Y | Y | Y | Y | Y | N | 66.7 | Moderate |
| Modi et al (64) | Y | Y | Y | Y | Y | Y | Y | Y | Y | 100 | Low |
| Gera et al (66) | Y | Y | Y | Y | Y | Y | Y | Y | Y | 100 | Low |
| Rajasekaran et al (70) | Y | N | N | Y | Y | Y | Y | Y | Y | 77.8 | Low |
| Patro et al (79) | Y | N | N | Y | Y | Y | Y | Y | Y | 77.8 | Low |
| Raman et al (88) | Y | Y | Y | Y | Y | Y | Y | Y | Y | 100 | Low |
| Orsolini et al (121) | Y | N | N | Y | Y | Y | Y | Y | Y | 77.8 | Low |
| Rao et al (117) | Y | N | N | Y | Y | Y | Y | Y | Y | 77.8 | Low |
| Shiva et al (98) | Y | Y | Y | Y | Y | Y | Y | Y | Y | 100 | Low |
| Bhattarai et al (123) | Y | N | N | Y | Y | Y | Y | Y | Y | 77.8 | Low |
| Doherty et al (122) | Y | N | N | Y | Y | Y | Y | Y | Y | 77.8 | Low |
| Kumar et al (105) | Y | N | N | Y | Y | Y | Y | Y | Y | 77.8 | Low |
| Barik et al (112) | Y | N | N | Y | Y | Y | Y | Y | Y | 77.8 | Low |
| Pahuja et al (102) | Y | N | N | Y | Y | Y | Y | Y | Y | 77.8 | Low |
| Mehrotra et al (107) | Y | N | N | Y | Y | Y | Y | Y | Y | 77.8 | Low |
| Bansal et al (124) | Y | Y | Y | Y | Y | Y | Y | Y | Y | 100 | Low |
| Mahadevan et al (119) | Y | N | N | Y | Y | Y | Y | Y | Y | 77.8 | Low |
| Biswas et al (91) | Y | N | N | Y | Y | Y | Y | Y | Y | 77.8 | Low |
| Ibrahim et al (101) | Y | N | N | Y | Y | Y | Y | Y | Y | 77.8 | Low |
| Khanna et al (85) | Y | N | N | Y | Y | Y | Y | Y | Y | 77.8 | Low |
| Manjunatha et al (109) | Y | N | N | Y | Y | Y | Y | Y | Y | 77.8 | Low |
| Jindal et al (46) | Y | N | Y | Y | Y | Y | Y | Y | Y | 88.9 | Low |
| Subramanian et al (31) | Y | Y | Y | Y | Y | Y | Y | Y | Y | 100 | Low |
| Mohanan et al (80) | Y | N | N | Y | Y | Y | Y | Y | Y | 77.8 | Low |

Q1 – Q9 indicates questions 1 to 9 as per the JBI appraisal tool.
Y - Yes, N – No, U – Unclear N/A – Not/Applicable
Scores less than 50% were rated as high risk of bias, 50 to 69% as moderate, and more than 70% as low risk of bias.

**S2-b Table: Quality assessment results of quantitative cohort studies using JBI checklist**

| **Study identification** | **Q1** | **Q2** | **Q3** | **Q4** | **Q5** | **Q6** | **Q7** | **Q8** | **Q9** | **Q10** | **Q11** | **% Score** | **Risk of Bias** |
| --- | --- | --- | --- | --- | --- | --- | --- | --- | --- | --- | --- | --- | --- |
| Bashingwa et al (27) | Y | U | Y | N | N | Y | Y | Y | Y | N | Y | 63.6 | Moderate |
| Anand et al (34) | Y | Y | Y | N | N | Y | Y | Y | N | N | Y | 63.6 | Moderate |
| Khan et al (45) | Y | Y | Y | N | N | Y | Y | Y | N | N | Y | 63.6 | Moderate |
| Birur et al (55) | Y | Y | Y | Y | Y | Y | Y | Y | N | N | Y | 81.8 | Low |
| Ravindran et al (77) | N | N | N | Y | Y | N | Y | Y | Y | N | Y | 54.5 | Moderate |
| Panda et al (84) | Y | Y | Y | Y | N | Y | Y | Y | Y | Y | Y | 90.9 | Low |
| Gupta et al (75) | Y | Y | Y | N | N | Y | Y | Y | N | N | Y | 63.6 | Moderate |
| Rout et al (74) | Y | Y | Y | Y | Y | Y | Y | Y | N | Y | Y | 90.9 | Low |
| Ganapathy et al (93) | Y | N | Y | N | N | Y | Y | Y | Y | N | Y | 63.6 | Moderate |
| Thakar et al (76) | Y | Y | Y | Y | Y | Y | Y | Y | Y | Y | Y | 100 | Low |
| Priya et al (106) | Y | Y | Y | N | N | Y | Y | Y | N | Y | Y | 72.7 | Low |
| Philip et al (97) | Y | Y | Y | Y | Y | Y | Y | Y | N | Y | Y | 90.9 | Low |
| Keeppanasserril et al (81) | Y | Y | Y | Y | Y | Y | Y | Y | N | Y | Y | 90.9 | Low |
| Deo and Singh (28) | Y | Y | Y | Y | Y | Y | Y | Y | Y | Y | Y | 100 | Low |
| Ganapathy et al (94) | Y | N | Y | N | N | Y | Y | Y | Y | N | Y | 63.6 | Moderate |

Q1 – Q11 indicates questions 1 to 11 as per the JBI appraisal tool.
Y - Yes, N – No, U – Unclear N/A – Not/Applicable
Scores less than 50% were rated as high risk of bias, 50 to 69% as moderate, and more than 70% as low risk of bias.

**S2-c Table: Quality assessment results of randomized controlled trial studies using JBI checklist**

| **Study identification** | **Q1** | **Q2** | **Q3** | **Q4** | **Q5** | **Q6** | **Q7** | **Q8** | **Q9** | **Q10** | **Q11** | **Q12** | **Q13** | **% Score** | **Risk of Bias** |
| --- | --- | --- | --- | --- | --- | --- | --- | --- | --- | --- | --- | --- | --- | --- | --- |
| Modi et al (39) | Y | N | Y | N | N | Y | N | Y | Y | Y | N | N | Y | 53.8 | Moderate |
| Carmichael et al (42) | Y | N | Y | N | N | Y | N | Y | Y | Y | Y | N | N | 53.8 | Moderate |
| Modi et al (43) | Y | N | Y | N | N | Y | N | Y | Y | Y | Y | Y | Y | 69.2 | Moderate |
| Patel et al (82) | Y | N | Y | N | N | Y | N | Y | Y | Y | Y | Y | Y | 69.2 | Moderate |
| Muke et al (21) | Y | N | Y | Y | Y | Y | Y | Y | Y | N | Y | N | Y | 76.9 | Low |
| Peiris et al (50) | Y | N | Y | N | Y | Y | Y | Y | Y | Y | Y | Y | Y | 84.6 | Low |
| Jain et al (120) | Y | Y | Y | N | N | Y | Y | Y | Y | Y | Y | Y | Y | 84.6 | Low |
| Bhattacharjya et al (23) | Y | N | Y | N | N | Y | N | Y | Y | Y | Y | Y | Y | 69.2 | Moderate |
| Shah et al (53) | Y | N | Y | N | N | Y | Y | Y | Y | Y | Y | Y | Y | 76.9 | Low |

Q1 – Q13 indicates questions 1 to 13 as per the JBI appraisal tool.
Y - Yes, N – No, U – Unclear N/A – Not/Applicable
Scores less than 50% were rated as high risk of bias, 50 to 69% as moderate, and more than 70% as low risk of bias.

**S2-d Table: Quality assessment results of qualitative studies using JBI checklist**

| **Study identification** | **Q1** | **Q2** | **Q3** | **Q4** | **Q5** | **Q6** | **Q7** | **Q8** | **Q9** | **Q10** | **% Score** | **Risk of bias** |
| --- | --- | --- | --- | --- | --- | --- | --- | --- | --- | --- | --- | --- |
| Lakshminarayanan et al (104) | Y | Y | Y | Y | Y | N | NA | Y | N | Y | 70 | Low |
| Babu et al (113) | N | Y | Y | Y | Y | N | N | N | Y | Y | 70 | Low |
| Srinidhi et al (13) | Y | Y | Y | Y | Y | N | N | Y | Y | Y | 80 | Low |
| Khan et al (32) | Y | Y | Y | Y | Y | N | N | Y | Y | Y | 80 | Low |
| Manjunatha et al (78) | Y | Y | Y | Y | Y | N | N | Y | Y | Y | 80 | Low |
| Modi et al (67) | Y | Y | Y | Y | Y | N | Y | Y | Y | Y | 90 | Low |
| Usmanova et al (20) | Y | Y | Y | Y | Y | N | N | Y | Y | Y | 80 | Low |
| Meher et al (90) | N | Y | Y | Y | Y | N | N | N | Y | Y | 70 | Low |
| Scott et al (22) | Y | Y | Y | Y | Y | N | N | Y | Y | Y | 80 | Low |
| Singh et al (29) | Y | Y | Y | Y | Y | N | N | Y | Y | Y | 80 | Low |
| Chattopadhyay et al (37) | Y | Y | Y | Y | Y | N | N | Y | Y | Y | 80 | Low |
| Muke et al (44) | Y | Y | Y | Y | Y | N | N | Y | Y | Y | 80 | Low |
| Birur et al (47) | Y | Y | Y | Y | Y | N | N | Y | Y | Y | 80 | Low |
| Abdel-All et al (48) | Y | Y | Y | Y | Y | N | N | Y | Y | Y | 80 | Low |
| Jarosławski et al (69) | Y | Y | Y | Y | Y | N | N | Y | Y | Y | 80 | Low |
| Prinja et al (60) | Y | Y | Y | Y | Y | N | N | Y | Y | Y | 80 | Low |
| Smith et al (65) | Y | Y | Y | Y | Y | N | N | Y | Y | Y | 80 | Low |

Q1 – Q10 indicates questions 1 to 10 as per the JBI appraisal tool.
Y - Yes, N – No, U – Unclear N/A – Not/Applicable
Scores less than 50% were rated as high risk of bias, 50 to 69% as moderate, and more than 70% as low risk of bias.

**S2-e Table: Quality assessment results of review studies using JBI checklist**

| **Study identification** | **Q1** | **Q2** | **Q3** | **Q4** | **Q5** | **Q6** | **Q7** | **Q8** | **Q9** | **Q10** | **Q11** | **% Score** | **Risk of Bias** |
| --- | --- | --- | --- | --- | --- | --- | --- | --- | --- | --- | --- | --- | --- |
| Naslund et al (49) | Y | Y | Y | Y | Y | Y | N/A | N/A | N/A | N/A | N/A | 100 | Low |
| Naslund et al (61) | Y | Y | N | Y | Y | Y | N/A | N/A | N/A | N/A | N/A | 83.33 | Low |
| Goel et al (71) | Y | Y | Y | N | Y | Y | N/A | N/A | N/A | N/A | N/A | 83.33 | Low |
| Angral et al (92) | Y | Y | Y | Y | N | Y | NA | NA | N | Y | NA | 55.55 | Moderate |
| Mahapatra et al (103) | Y | Y | Y | N | Y | Y | N/A | N/A | N/A | N/A | N/A | 83.33 | Low |

Q1 – Q11 indicates questions 1 to 11 as per the JBI appraisal tool.
Y - Yes, N – No, U – Unclear N/A – Not/Applicable
Scores less than 50% were rated as high risk of bias, 50 to 69% as moderate, and more than 70% as low risk of bias.

**S2-f Table: Quality assessment results of quasi-experimental studies using JBI checklist**

| Study identification | **Q1** | **Q2** | **Q3** | **Q4** | **Q5** | **Q6** | **Q7** | **Q8** | **Q9** | **Q10** | **% Score** | **Risk of Bias** |
| --- | --- | --- | --- | --- | --- | --- | --- | --- | --- | --- | --- | --- |
| Chang et al (110) | Y | N | Y | NA | Y | Y | Y | NA | Y | N | 60 | Moderate |
| Dhanasekaran et al (100) | Y | Y | Y | Y | Y | Y | Y | NA | Y | N | 80 | Low |
| Nethan et al (116) | Y | N | Y | Y | Y | Y | Y | NA | Y | N | 70 | Low |
| Thukral et al (115) | Y | N | Y | Y | Y | Y | Y | NA | Y | N | 70 | Low |
| Dandge et al (51) | Y | N | Y | Y | Y | Y | Y | NA | Y | N | 70 | Low |
| Balasubramaniam et al (99) | Y | N | Y | Y | Y | Y | Y | NA | Y | N | 70 | Low |
| Gautam et al (108) | Y | N | Y | Y | Y | Y | Y | NA | Y | N | 70 | Low |
| Sagi et al (111) | Y | N | Y | Y | Y | Y | Y | NA | Y | N | 70 | Low |
| Hariprasad et al (118) | Y | N | Y | Y | Y | Y | Y | NA | Y | N | 70 | Low |

Q1 – Q10 indicates questions 1 to 10 as per the JBI appraisal tool.
Y - Yes, N – No, U – Unclear N/A – Not/Applicable
Scores less than 50% were rated as high risk of bias, 50 to 69% as moderate, and more than 70% as low risk of bias.

**S2-g Table: Quality assessment results of mixed-methods studies using MMAT checklist**

| **Study identification** | **Q1** | **Q2** | **Q3** | **Q4** | **Q5** | **Comments** |
| --- | --- | --- | --- | --- | --- | --- |
| Harding et al (38) | Y | Y | Y | C | C | The study utilized a qualitative and quantitative design to address the research questions. The different components were effectively integrated to answer the research question. |
| Schierhout et al (30) | Y | Y | Y | C | Y | A mixed-method design adequately used to address the research question. |
| Charanthimath et al (25) | Y | Y | Y | C | C | The output measures used were not adequate |
| Suryavanshi et al (41) | Y | Y | C | C | C | Different methods were integrated to answer the research question. |
| Shah et al (40) | Y | Y | C | C | C | An in-depth qualitative analysis of ASHA and beneficiaries was utilized in the study |
| Thukral et al (68) | Y | Y | C | C | C | The objective measurement utilized OSCE interviews. |
| Maulik et al (62) | Y | Y | Y | Y | C | The components of the intervention were assessed adequately. |
| Bhatt et al (52) | Y | Y | Y | C | C | The acceptability of the application was assessed with mixed methods |
| Ramanathan et al (114) | Y | Y | Y | Y | C | Mixed methods gave an adequate evaluation of the acceptability of the study |
| Gupta et al (24) | Y | Y | Y | Y | Y | A qualitative approach helped establish the acceptance testing of the sample population. |
| Ilozumba et al (57) | Y | Y | Y | Y | C | The components of the intervention were assessed adequately. |
| Sharma et al (56) | Y | Y | Y | Y | C | The different components were effectively integrated |

^a^Q1 – Q5 indicates questions 1 to 5 as per the MMAT appraisal tool.
Y – Yes, C – Can’t tell
