# Supplementary material for: Barriers and facilitators for the use of telehealth by healthcare providers in India—A systematic review
Source: PLOS Digit Health. 2024 Dec 6;3(12):e0000398. doi: 10.1371/journal.pdig.0000398 (PMC11623477; doi:10.1371/journal.pdig.0000398)
Supplement: Attachment — Submitted filename: PDIG-D-23-00391_reviewer 4 _JULIAN BORGES.pdf [file pdig.0000398.s013.pdf]

Barriers and Facilitators for the Use of Telehealth by Healthcare Providers (HCP) in India - A Scoping Review

Reviewer Recommendation and Comments for Manuscript Number PDIG-D-23-00391R3
Author: Julian Borges

Summary: This systematic review examines the barriers and facilitators to telehealth implementation in India. The study provides a comprehensive overview of the current state of telehealth in India, identifying key challenges and opportunities. The review is well-structured and offers valuable insights for policymakers and healthcare providers.

Major issues:

1. Single database search: Regarding your methodology, while your search strategy is comprehensive, it's limited to PubMed and that alone may have led to missing relevant studies. (risk of bias). I'd encourage you to consider expanding your search to include other databases, or provide a rationale for their exclusion in the limitations section explaining this financial situation to the readers, (it was explained to the reviewers only), clarifying in the narrative to the readers would be appropriate.

2. Lack of meta-analysis: While not always necessary, a quantitative synthesis of results could provide additional insights and robustness.

Minor issues:

1. Language restriction: Including only English language studies may introduce language bias.

2. Limited critical appraisal: A more in-depth analysis of study outcomes and risk of bias could strengthen the review.

3. Incomplete geographic data: Information on study locations was missing for some studies.

Other comments: The review provides a good foundation for understanding telehealth implementation in India. Consider expanding on policy implications and future research directions.

Figures and tables: Clearly presented and correctly labeled? The figures and tables appear to be clearly presented and correctly labeled, providing a good visual summary of the findings.

Methods: Sufficiently detailed? The methods are generally well-described. However, more detail on the quality assessment process could be beneficial.

Study Design: Appropriate for the question? The systematic review design is appropriate for the research question.

Barriers and Facilitators for the Use of Telehealth by Healthcare Providers (HCP) in India - A Scoping Review

Any experiments missing? Not applicable for a systematic review.
Statistics: Statistical analyses sound? No statistical analyses were performed, which is noted in the manuscript. An explanation for this decision could be helpful.

Results and data: Support conclusions? The results appear to support the conclusions drawn in the review.

All data accessible? The data presented in the review seems accessible. Consider a Data Availability Statement at the start and if there's any additional data that could be included in supplementary materials also state.

References: Anything missing? The references appear comprehensive, but a cross-check with other databases could ensure no key studies were missed.

Title: Appropriate and informative? The title appears appropriate and informative, clearly stating the focus of the review.

User preferred language: English. The review is written in clear, professional English.

2. Does this manuscript meet PLOS Digital Health's [publication criteria](#)? Is the manuscript technically sound, and do the data support the conclusions? The manuscript must describe methodologically and ethically rigorous research with conclusions that are appropriately drawn based on the data presented.

This manuscript largely meets PLOS Digital Health's publication criteria, but there are some areas that may need improvement.

1. Technical soundness: The review seems to be technically sound overall. The systematic review methodology is appropriate for the research question, and the methods are generally well-described. However, there are some limitations:

    - The use of only one database (PubMed) for the literature search is a significant limitation that may affect the comprehensiveness of the review. (already addressed in the beginning of this report)
    - The lack of a meta-analysis, while not always necessary, could have provided additional insights.

2. Methodological rigor: The systematic review approach is methodologically appropriate. However, there are areas for improvement:

    - Expanding the literature search to include more databases would enhance rigor.
    - More detail on the quality assessment process could strengthen the methodology.
    - The lack of statistical analyses is noted, though an explanation for this decision could be helpful.

Barriers and Facilitators for the Use of Telehealth by Healthcare Providers (HCP) in India - A Scoping Review

4. Have the authors made all data underlying the findings in their manuscript fully available (please refer to the Data Availability Statement at the start of the manuscript PDF file)? NO

The PLOS Data policy requires authors to make all data underlying the findings described in their manuscript fully available without restriction, with rare exception. The data should be provided as part of the manuscript or its supporting information, or deposited to a public repository. For example, in addition to summary statistics, the data points behind means, medians and variance measures should be available. If there are restrictions on publicly sharing data—e.g. participant privacy or use of data from a third party—those must be specified.

The manuscript does not appear to contain a Data Availability Statement at the start, which would typically specify how the underlying data can be accessed. (Easy fix)

Final comments: Almost good to go!
