# Supplementary material for: Barriers and facilitators for the use of telehealth by healthcare providers in India—A systematic review
Source: PLOS Digit Health. 2024 Dec 6;3(12):e0000398. doi: 10.1371/journal.pdig.0000398 (PMC11623477; doi:10.1371/journal.pdig.0000398)
Supplement: Attachment — Submitted filename: Response to reviewers comments.docx [file pdig.0000398.s012.docx]

**Responses to Reviewer Comments:**

Manuscript Title: Barriers and Facilitators for the Use of Telehealth by Healthcare Providers (HCP) in India - A Systematic Review

Manuscript ID: PDIG-D-23-00391

**Reviewer #1**

This study is a comprehensive systematic review of barriers and facilitators to telehealth use by healthcare practitioners in India. This study provides interesting findings with the potential to inform healthcare delivery in India.

**General Comments: This study was originally presented as a scoping review but is now being positioned as a systematic review. This should be acknowledged as a limitation and deviation from the original protocol (with justification). However, just suggesting this review is now a systematic review because it did not follow scoping review guidelines is problematic. For example, while scoping reviews do not assess risk of bias, systematic reviews are generally required to assess and report risk of bias. While the risk of bias was “N/A” when it was a scoping review, you will need to justify why not risk a bias was done and acknowledge this as a limitation.**

Response: We thank the reviewer for this comment. We have added the risk of bias assessment in Supplementary Tables 2a to 2g. We have also updated the methods section to include the risk of bias assessment, and have also added a paragraph on our assessment in the results section.

**Comment: “At Level 1, the articles were screened based on the title and abstract. The articles included by any one reviewer at Level 1 screening were moved to Level 2.” What guided the authors screening approach?**

Response: The inclusion and exclusion criteria that guided screening and selection have been mentioned in the “Screening and Selection” section of Methods -

“Inclusion criteria included studies conducted in India and reported in English that focused on the use of telehealth by healthcare providers. Studies only evaluating clinical outcomes but not related to HRH cadre or management strategies or practices and study protocols, editorials, viewpoints, commentaries, letters, and correspondences were excluded.”

**Comment: “The full text of all the articles in Level 2 was reviewed independently by two reviewers. After the full-text screening, articles were finally excluded or included only if both reviewers were in agreement.” Can the authors provide inter rater reliability score for level 2 screening?**

Response: We thank the reviewer for this comment. Following the standard well-established protocols under the Cochrane guidelines for conducting evidence synthesis, two investigators filled in the study information and compiled that to have a more accurate and complete dataset. Since these were not ‘scored’ items but actual data of various types extracted from different parts of the paper a simple inter-rater assessment is neither possible nor required.

**Reviewer #3: My comments are adequately addressed**

Response: We thank the reviewer for their valuable input.

**Reviewer #4:**

**Comment: The authors indicated that they choose only Pubmed because “it is open access”. PubMed is not necessarily an open-access databases because it only provides free access to the title and abstract and not full text. Full texts are available in PubMed ONLY if they are published as such in their respective journals. If a paper is not published as open-access, PubMed does not index that paper as a full-text in its database. If cost is indicated as a limitation, I am wondering how the authors retrieved the full text for articles that have free access to only titles and abstract in PubMed? I just searched for some of the included articles and beyond the title and abstracts, PubMed does not provide access to the full text? So how did the authors access the full text if cost was indicated as a limitation?**

Response: We thank the reviewer for their comment. Pubmed is an open-access search database, i.e., there is no fee or account registration required to search the database. In contrast, databases like Embase and Web of Science require fee-based subscriptions to run searches. Pubmed can index articles that are not open-access. However, those are two different things. We have mentioned the former and not the latter in our manuscript.

As the reviewer has pointed out, the full texts that were not available on PubMed were accessed by authors using the institution’s access to the journal. The authors from Christian Medical College, Vellore, India helped in retrieving some of these articles. Despite all efforts we were not able to retrieve the full text of 23 articles. The same has been mentioned in the PRISMA flowchart.

**Comment: They also stated that one of the reasons for choosing only PubMed is because it is “a specialized and curated database”. I think this is the more reason why more databases are needed. Digital health is an interdisciplinary field and using a “specialized” database may not be able to capture the diverse literature in the field**

Response: We thank the reviewer for their comment. By specialized, we meant that Pubmed had literature specifically about health and medicine, unlike a database like Google Scholar which contains articles across various disciplines, which can be irrelevant to the research question(s). We agree with the reviewer and this is why the inclusion of only PubMed has been added as a limitation. However, this study is the largest review on telehealth use in India as broad inclusion criteria were used to select studies.

**Comment: Assuming without admitting that only PubMed was used because it is open-access, I think I think there are co-authors with affiliations to Columbia, Duke university, Harvard, UCL etc that might have had access to all databases to help expand the search. Therefore, cost cannot be an adequate justification when other avenues weren’t explored**

Response: We understand the reviewer's concern and hope that as a fellow researcher, they would understand the complexity and challenges of this research. As mentioned previously, the work for this review was conducted as part of the larger body of work under the Lancet Citizens’ Commission by volunteers. While some investigators may have had access to the privileged databases through their individual institutional affiliations, such access (to Web of Science, Embase, etc.)) was not possible to share with the entire team of investigators due to stringent regulatory policies of the proprietary databases. Hence, as a group of researchers working across institutions and countries on a project that did not have any direct funding support, we had to choose what would work best for the entire team, especially the researchers based in India.

**Comment: The authors also decided to revert to a systematic review based on my previous comment. While this is laudable, I think they should go beyond just changing the name from scoping review to a systematic review by appraising the quality of the evidence. Without a quality appraisal of the literature, it still does not qualify as a systematic review, in my view.**

Reviewer: We thank the reviewer for this comment. We have added the risk of bias assessment. Detailed assessment is presented in the supplementary file. We have also updated the methods section to include the risk of bias assessment, and have also added a paragraph on our assessment in the results section.

**Reviewer #5:**

**The authors have thoughtfully considered all comments and have addressed them where possible. While there are some limitations in the review's methodology, primarily the use of only one database for searching, the authors have explained in detail why this is the case. The search strategy was also previously registered. This review provides useful look at how Indian healthcare is using mHealth applications.**

Response: We thank the reviewer for their valuable inputs.
