# Supplementary material for: Barriers and facilitators for the use of telehealth by healthcare providers in India—A systematic review
Source: PLOS Digit Health. 2024 Dec 6;3(12):e0000398. doi: 10.1371/journal.pdig.0000398 (PMC11623477; doi:10.1371/journal.pdig.0000398)
Supplement: Attachment — Submitted filename: 2_Sep_24_Response to reviewers comments.docx [file pdig.0000398.s014.docx]

**Responses to Reviewer Comments:**

Manuscript Title: Barriers and Facilitators for the Use of Telehealth by Healthcare Providers (HCP) in India - A Systematic Review

Manuscript ID: PDIG-D-23-00391

**Reviewer #3: My comments are addressed**

Response: We thank the reviewer for their valuable input.

**Reviewer #4:**

**Comment: Many thanks for the opportunity to re-review this manuscript. While the authors might have addressed almost all comments, I think the substantive concern remains unaddressed and I don’t think the explanations helps either. The use of a single database is a huge flaw that cannot simply be discounted as a study limitation. Without a robust literature search, I see this as someone trying to put up a house on a week foundation. The issue of cost would have been totally acceptable if it probably led the authors to limit the geographical scope of the review or to tie it to specific types of digital health technologies. However, limiting the methodological scope, with all due respect, falls short of the standards required for systematic reviews. In systematic reviews, searching is one thing and retrieving the literature is another thing. You can conduct searchers in PubMed but cannot retrieve the literature from PubMed, unless they are open access. I don’t think all the 2944 articles were open access and it would be erroneous for institutions to subscribe to individual journals (where you were able to retrieve this tone of literature) and not subscribe to publishing companies where they can have access to many journals as possible.**

**I want to author to take this one main criticism in good faith. I am not being critical for the sake of it but to make sure that the eventual publication will be robust enough and meet the rigor expected of a systematic review.**

Response: We once again agree with the reviewer and take the criticism in good faith. We have accepted and declared the use of a single database PubMed as a limitation of the study. However, the reviewer is suggesting re-doing the entire study which is not feasible. We will keep the reviewer’s valuable suggestions in mind while conducting reviews in the future.

**Reviewer #6:**

**Comment: Thank you for the opportunity to review your manuscript on telehealth use by healthcare providers in India. I found your work to be a valuable contribution to the field, addressing a timely and important topic. Your systematic approach and comprehensive analysis of 106 studies provide a solid foundation for understanding the landscape of telehealth in India.**

**I particularly appreciated your thorough examination of facilitators and barriers across mHealth, telemedicine, and tele-education. This multi-faceted approach offers valuable insights for both practitioners and policymakers.**

Response: We thank the reviewer for their positive comments.

**Comment: Firstly, I noticed the absence of a Data Availability Statement. As per PLOS policy, this is a crucial element that needs to be addressed.**

Response: We would like to bring to the reviewer’s notice that the data file has been uploaded along with the submission. We have added a data availability statement in the manuscript.

**Comment: Regarding your methodology, while your search strategy is comprehensive, it's limited to PubMed. I'd encourage you to consider expanding your search to include other databases like Embase or Web of Science, or provide a rationale for their exclusion in the or clear statement explaining, i know you have alrady explained this, but clarify in the narrtive.**

Response: We agree with the reviewer and have acknowledged this as a major limitation. We would like to bring to the reviewer’s notice that the inclusion of only one database has been included as a limitation of our study and the following statement has been already mentioned in the “Strengths and Limitations” section-

Line 583-587 - *Finally, due to lack of funding for the project we could not access databases like EMBASE and Scopus. This might have led to the exclusion of a few studies available on these databases. However, the large volume of papers included adds to the comprehensiveness of our findings. Also, our strategy is replicable in other LMICs and resource-limited settings.*

That said, we would like to provide some context for our choice. As described in the review protocol cited in the paper, this review is part of a larger set of reviews synthesized to inform the ongoing work of the Lancet Commission for Reimagining India’s Health System. The choice for Pubmed was based on the fact that it is an open-access, specialized, and curated database. We wanted to consider literature that passed the peer review check as the minimum requirement for scientific credibility and integrity to use them to inform the Commission confidently. Hence, sources of gray literature, such as Google Scholar, were avoided. Also, we wanted to ensure that the search is replicable for free around the world (open access). Hence, cost-prohibitive sources such as Web of Science and Scopus were avoided.

We have updated the manuscript and explained this context in the limitations section.

**Comment: The risk of bias assessment could benefit from more detailed explanation. A summary table of these assessments would be a valuable addition to your manuscript.**

Response: We thank the reviewer for their comment and would like to bring to the reviewer’s notice that detailed risk of bias summary tables have been uploaded with the manuscript as Supplementary Tables 2(a-g). A summary has been added to the results section as text. We believe that together these serve the purpose in explaining the risk of bias of the studies that we have included.

**Comment: Your analysis, while thorough, could be deepened in certain areas. For instance, a more quantitative synthesis of findings, where possible, would enhance the impact of your work. Additionally, further exploration of differences between public and private providers, and more discussion on regional variations would provide richer insights.**

**Response:** We agree with the reviewer that there is a need for further evidence synthesis and thank them for the comment. We attempted a regional assessment of facilitators and barriers, however, we did not find any specific pattern to report. We have mentioned this as a future recommendation for studies.

While extracting data, public and private differentiation was not made. We have added this as a future recommendation in the “Conclusion” section.

We have also already mentioned this in the limitations section:

Line 565-567: Firstly, facilitators and barriers could not be differentially studied between private and public providers as the distinction between the type of provider could not be made using extracted data.

**Comment: I'd also suggest expanding your discussion of limitations to include potential language bias and the lack of grey literature inclusion.**

**Response:** We thank the reviewer for their comment. We have expanded the limitation section to include the suggested limitations. However, as only the facilitators and barriers in India were being studied, English being the commonest language, the risk of language bias is unlikely in our opinion.

**Comment: Your policy implications section could be more specific, offering concrete recommendations based on your findings.**

Response: We thank the reviewer for their comment. We have expanded the policy recommendation section.

**Comment: Lastly, some of your figures (particularly Figures 2, 5, and 8) could be improved for clarity. Consider using color gradients or larger font sizes for state names.**

Response: We thank the reviewer for their comment. We have changed the color gradient for easier interpretation and added a color legend.

**Reviewer #7:**

**Comment: The manuscript seems to address all previous reviewer comments. It presents an interesting work pertinent to the Indian healthcare system. Coming from a background where I also work in telemedicine for LMICs, the findings of this review seem to align greatly with the challenges and opportunities we experience. I think the work is beneficial not only for Indian contexts, but in general for developing healthcare systems. I do not have any comments regarding further improvements at this time.**

Response: We thank the reviewer for their kind comment.
