# Supplementary material for: Barriers and facilitators for the use of telehealth by healthcare providers in India—A systematic review
Source: PLOS Digit Health. 2024 Dec 6;3(12):e0000398. doi: 10.1371/journal.pdig.0000398 (PMC11623477; doi:10.1371/journal.pdig.0000398)
Supplement: Attachment — Submitted filename: Response Reviewer Comment.docx [file pdig.0000398.s010.docx]

**Responses to Reviewer Comments:**

Manuscript Title: Barriers and Facilitators for the Use of Telehealth by Healthcare Providers (HCP) in India - A Scoping Review

Manuscript ID: PDIG-D-23-00391

Journal Requirements:

1. Please provide separate figure files in .tif or .eps format only and remove any figures embedded in your manuscript file. Please also ensure that all files are under our size limit of 10MB.

For more information about figure files please see our guidelines:

https://journals.plos.org/digitalhealth/s/figures

https://journals.plos.org/digitalhealth/s/figures#loc-file-requirements

Response: Images embedded in the manuscript have been removed and images in the appropriate format have been submitted

2. Some material included in your submission may be copyrighted. According to PLOS’s copyright policy, authors who use figures or other material (e.g., graphics, clipart, maps) from another author or copyright holder must demonstrate or obtain permission to publish this material under the Creative Commons Attribution 4.0 International (CC BY 4.0) License used by PLOS journals. Please closely review the details of PLOS’s copyright requirements here: PLOS Licenses and Copyright. If you need to request permissions from a copyright holder, you may use PLOS's Copyright Content Permission form.

Potential Copyright Issues:

Figs 2, 5 7 8: please (a) provide a direct link to the base layer of the map (i.e., the country or region border shape) and ensure this is also included in the figure legend; and (b) provide a link to the terms of use / license information for the base layer image or shapefile. We cannot publish proprietary or copyrighted maps (e.g. Google Maps, Mapquest) and the terms of use for your map base layer must be compatible with our CC-BY 4.0 license.

Response: Datawrapper (an online platform) was used to make the maps. The website mentions that the creator has the copyright to all the maps created using the website - <https://www.datawrapper.de/faq#can-i-use-my-charts-or-maps-in-a-commercial-product-or-publication>

Reviewer #1:

This study is a comprehensive scoping review of barriers and facilitators to telehealth use by healthcare practitioners in India. This study provides interesting findings with the potential to inform healthcare delivery in India.

General Comment: The use of acronyms is inconsistent and should be double-checked. Some acronyms are never defined, some are used only once, and some are defined multiple times. Additionally, make sure citations are used appropriately, there were a few sentences that were missing citations. The discussion section was mostly a repetition of the findings and would be strengthened by contextualizing the findings within the existing literature. The authors should also consider reviewing the discussion section to improve clarity and grammar.

Response: We thank the reviewer for this comment. We have made the necessary corrections to the use of acronyms. We have also revised the discussion.

Specific Comments:

Comment: On Page 4, when the authors discuss the costs of healthcare, is that to individuals or system costs?

Response: The cost of healthcare mentioned on Page 4 refers to the out-of-pocket expenditure incurred by individuals while seeking care. We have edited the statement to bring this out clearly.

Comment: On Page 5, access to health services in what way, affordability? Accessibility?

Response: eSanjeevani is a government-led telemedicine service that has improved access in terms of both affordability and accessibility. We have edited the statement to clarify this.

Comment: Page 5 lines 105 to 109 could be reworded for clarity.

Response: The lines have been reworded for better clarity.

Comment: Page 6, do the authors have a justification for the timeframe of the search?

Response: This review was part of a larger evidence synthesis work on human resources for health for universal health coverage in India. That project aimed to study the available literature over the past two decades. However, despite following the same timeframe for the search, the first paper fitting the inclusion criteria was published in 2009. This excludes the possibility of missing out on any relevant literature published before 2001. The last search was performed on 17th February 2022 and following that review and analysis of the identified articles was commenced.

Comment: Page 7, definitions are provided for mHealth and telemedicine in the background, but not for tele-education, how is this a distinct category for the authors.

Response: As tele-education engages only the healthcare providers and not the patients, it was considered as a separate category as barriers and facilitators associated with its use were anticipated to be different.

Comment: Page 7, can more description be provided on the analysis technique.

Response: The data analysis section has been updated to describe the analysis technique in detail.

Comment: Page 19 line 406, are the authors referring to healthcare delivery?

Response: We are referring to all the HRH practices, including but not limited to healthcare delivery. These practices have been defined in Supplement 4.

Comment: Page 20 line 427 -429, “An understanding of the facilitators and barriers emphasizes the need for understanding the same at multiple dimensions especially focusing on the facilitators and barriers related to Human resources for health (HRH), Infrastructure, and Technology” What are the authors referring to when the describe “understanding the same”

Response: We are referring to the “need for policy modifications” as “the same”. The statement has been reframed for a better understanding of the same.

Comment: Page 20 line 432, do the authors mean “roadblock”?

Response: The word “roadblock has been replaced by “barrier”.

Comment: Page 20 line 435-436. This sentence is clumsy. The conclusion they are drawing here is that because women have "lesser access" to mobile phones and technology, increasing network coverage must also consider gender equality. I do not see how these two things are related as the authors have described it. Gender equity is a consideration in technology use and access. How do the authors suggest gender equity be considered in network coverage increases? How are the authors defining network coverage? Does that include access to devices, or broadband Internet and cell coverage as it is typically used?

Response: We thank the reviewer for this comment. We agree with the reviewer's view and have therefore removed the statement and revised the discussion section.

Comment: Page 21 451-453, “Initiatives like the ‘G-20 Digital Innovation Alliance’ show promise in encouraging digital health startups by providing grants, sponsorship, and collaboration opportunities in order to strengthen the telehealth scenario”. This feels out of place, did the review identify start ups as a facilitator. I am not disagreeing with this point, just need a little more justification for how it fits within the discussion of barriers and facilitators.

Response: We agree with the reviewer’s comment that this statement is out of context in this paper and therefore we have removed it and modified the section.

Reviewer #2:

The manuscript's language should be clear, correct, and unambiguous. To enhance readability, eliminate unnecessary content while ensuring that the paper maintains clarity. Major modifications are required in terms of grammar, punctuation, coordinating conjunctions, articles, and spaces between words. E.g.:

Comment: The manuscript was checked for plagiarism, and it found a high use of generative AI without any declaration of such use.

Response: The authors would like to declare that AI was not used to prepare this manuscript. The entire manuscript was written by the first two authors and reviewed by the co-authors.

Also, there is a huge difference in the writing pattern in different sections or paragraphs.

Response: The manuscript was co-written by the first two authors. The first author has reviewed the entire manuscript again and made appropriate changes to address the reviewer's concern.

Additionally, some areas are not properly cited.

Response: All statements have been double-checked to ensure proper citations and corrections, where needed, have been made.

Comment: It’s noted that the used map figures are all subjected to copyright, and can not be used without a written permission.

Response: Datawrapper (an online platform) was used to make the maps. The website mentions that the creator has the copyright to all the maps created using the website - <https://www.datawrapper.de/faq#can-i-use-my-charts-or-maps-in-a-commercial-product-or-publication>

Comment:

Please clarify the abbreviation (HCPs) in first mention and not in the title.
Please clarify the abbreviation (HRH) just in first mention and not every time you mention.
Please clarify the abbreviation (mHealth) in first mention.
In page 5, line 109, what do you mean by ‘HR’?
And there is many more, make sure to do proofreading.

Response: We would like to thank the reviewer for pointing out this error. All the abbreviations have now been clarified in the first mention.

Comment: The manuscript claims to be a comprehensive analysis of telehealth in the Indian health system, yet a critical examination reveals several methodological and conceptual shortcomings that cast doubt on the validity and generalizability of the findings. While the effort to explore the facilitators and barriers of telehealth is commendable, the study lacks the depth and rigor necessary for drawing robust conclusions.

Response: We understand the concern of the reviewer. We agree that the study lacks further depth and therefore, we have mentioned all significant limitations towards the end of the manuscript and have also been cautious in terms of making interpretations and reaching conclusions. This scoping review aimed to provide a list of all the facilitators and barriers as per the available published literature in India. The generated evidence will guide subsequent projects to look deeply at the region-specific facilitators and barriers.

Comment: Please clarify the omission of telepharmacy in this study, while it is an important point for consideration, as a significant component of telehealth and contributing to overall healthcare efficiency. Its exclusion from the discussions in this manuscript leaves a notable gap in the comprehensiveness of the review. Please redo the study work and add telepharmacy.

Response: Telepharmacy papers were not excluded from the search strategy and inclusion criteria. As the aim of the review was to study the use of telehealth by healthcare providers, studies focusing on the patient experiences with telehealth were excluded. The articles included at the final level did not have any paper on barriers and facilitators associated with the use of telepharmacy by healthcare providers. We have added this as a limitation of this review.

Comment: The manuscript tends to overly emphasize the positive impacts of telehealth without providing a balanced view. A more critical approach would involve exploring potential drawbacks, ethical considerations, and challenges faced by both healthcare providers and patients in adopting telehealth technologies.

Response: We have revised the discussion to clearly bring out a more balanced view on the impacts of telehealth.

Comment: The review does not adequately explore cultural factors that might influence the adoption and acceptance of telehealth in India.

Response: Sociocultural barriers like poor literacy, stigma related to the disease, stigma related to technological intervention, and lack of human touch in the use of technology have been mentioned in the results section. The discussion has been revised to bring focus to these factors.

Comment: State which states were included in the review and if there were any reasons for the concentration in those particular regions. The study acknowledges a concentration of telehealth interventions in only 8 states/union territories, highlighting a significant regional bias.

Response: The literature search was done to identify studies on the use of telehealth in India. States and union territories in which the studies were conducted were then analyzed to assess the regional distribution of evidence on telehealth use. The exclusion criteria did not particularly exclude any state. The discussion section has been revised to explain this finding.

Comment: Discuss any potential differences or challenges related to the utilization of telehealth in the private sector, especially considering its accessibility to different socioeconomic strata.

Response: The objective of the paper was to describe the barriers and facilitators of the use of telehealth from the healthcare provider’s end. A distinction between facilitators and barriers in the public and private sectors could not be made using extracted data. We have added this as a limitation of our study.

Comment: Mere mention of the G-20 Digital Innovation Alliance is not sufficient. A more critical discussion on the inadequacy of the current funding levels (2.1% of GDP) and potential consequences is warranted.

Response: We agree with the reviewer’s comment. Keeping the comment of both the reviewers in mind we have modified the section.

Comment: Is there any comparative analysis or synthesis of findings across different studies, as identifying common themes or emerging patterns?

Response: We have reported the facilitators and barriers by doing a thematic analysis across all selected studies and reported the frequency of the identified themes in the results section. However, the identification of regional or cadre-specific patterns was out of the scope of this paper. We would look at more specific patterns in our upcoming reviews and we have added this as a future recommendation as well.

Comment: The review provides a surface-level overview without a critical analysis of the study outcomes. A deeper examination of the methodologies, results, and implications of the included studies is essential for a more meaningful contribution.

Response: We agree with the comment and our future work is going to look into that but currently it is out of the scope of this paper due to the large volume of the papers included. We will be doing a region-specific and cadre-specific analysis in our upcoming review.

Comment: Relying solely on PubMed for the search strategy raises concerns about the comprehensiveness of the literature review. The exclusion of other databases like Scopus and EMBASE might result in a biased representation of the available evidence.

Response: We agree with the reviewer’s comment. As this project did not receive any funding and the team of researchers was based in India we did not have access to other paid databases. However, the large volume of papers included adds to the comprehensiveness of our findings. Also, our strategy is replicable in other LMICs and resource-limited settings. The inclusion of only PubMed has also been mentioned as a limitation of this review.

Comment: Certain claims, such as the strong impact of telehealth on patient care and the assertion that it reduced travel constraints, lack concrete evidence or references. These statements should be supported by robust data to enhance the credibility of the review.

Response: We have added a reference to support these claims.
