# Supplementary material for: Barriers and facilitators for the use of telehealth by healthcare providers in India—A systematic review
Source: PLOS Digit Health. 2024 Dec 6;3(12):e0000398. doi: 10.1371/journal.pdig.0000398 (PMC11623477; doi:10.1371/journal.pdig.0000398)
Supplement: Attachment — Submitted filename: Response Reviewer Comment.docx [file pdig.0000398.s011.docx]

**Responses to Reviewer Comments:**

Manuscript Title: Barriers and Facilitators for the Use of Telehealth by Healthcare Providers (HCP) in India - A Scoping Review

Manuscript ID: PDIG-D-23-00391

Reviewer #3:

**Comment: The digital library considered in the study is only PubMed. Large number of telehealth solutions are proposed on other database. Instead of relying on a single database, it would have been better to shirink the scope and assess studies from everywhere in formulated scope.**

Response: We agree with the reviewer and have acknowledged this as a major limitation. That said, we would like to provide some context for our choice. As described in the review protocol attached in the supplement, this review is part of a larger set of reviews synthesized to inform the ongoing work of the Lancet Commission for Reimagining India’s Health System. The choice for Pubmed was based on the fact that it is an open-access, specialized, and curated database. We wanted to consider literature that passed the peer review check as the minimum requirement for scientific credibility and integrity to use them to inform the Commission confidently. Hence, sources of gray literature, such as Google Scholar, were avoided. Also, we wanted to ensure that the search is replicable for free around the world (open access). Hence, cost-prohibitive sources such as Web of Science and Scopus were avoided.

**Comment: I think telemedicine and tele-education should be part of the keyword list**

Response: We thank the reviewer for their comment. The terms were not a part of the search strategy as the search strategy was created for broader review(s) focused on human resources for health and human resource management practices in the context of Universal Health Coverage (for reference, see the protocol link - https://osf.io/kq3u9). The original plan was that the broad search would let us have multiple reviews aligning to different inter-related issues. It was decided that one of these reviews would focus on telehealth literature gathered this way. However, despite not including these terms we were still able to extract information from a large volume of articles. Our review persists to be the single largest review on telehealth in India.

We have added this explanation in the limitation section of the manuscript.

**Comment: Placement of references - putting the references at the middle or end of statements increases readability of the paper. For instance, mHealth, on the other hand, refers to applications or programs used on smartphones or tablets (1) preferebale than (1) mHealth, on the other hand, refers to applications or programs used on smartphones or tablets**

Response: We would like to inform the reviewer that the references have been placed at the middle or end of the statement. “(1)” is also the reference for the previous statement. Since the same paper has been used to write multiple non-overlapping statements, we have cited it multiple times.

**Comment: Acronyms need to be defined at their first in-text appearance. Take HRH, it is defined in its second appearance at page 6, though the acronym appeared at page 5. There are also undefined acronyms...e.g. LMICs**

Response: We thank the reviewer for this comment. We have made the necessary changes. We would like to bring to the reviewer’s notice that LMIC has been defined in line 447 in the discussion section.

**Comment: All Inclusion and exclusion criteria should be explicitly stated instead of mentioning some of them and indicating presence of more criteria with word like "like", "included", etc**

Response: We would like to inform the reviewer that inclusion and exclusion criteria have been explicitly mentioned in lines 136-140.

**Comment: Where is search string/query?**

Response: The search query has been described in complete detail in the S1 panel, which is a part of the supplementary material.

**Comment: There are many non-mHealth web-based and desktop software as well as websites ehealth solutions/interventions that are reported in literature. Same is true for hardware solutions. The remove didn't address reports on such solution. Hence, this needs to be mentioned either in the introduction or limitation of the study section.**

Response: We thank the reviewer. We have added this as a limitation of the study.

**Comment: Some reviews results could be better presented using tables/figures than as a paragraph. Example, roles of interventions.**

Response: We thank the reviewer for the comment. However, the manuscript currently has three tables and ten figures for the result section. Hence, we believe that the remaining findings would be better presented as text.

**Comment: I expected more technical future works than proposing other scoping review. For instance, what should be done to exploit the advantages of telehealth**

Reseponse: We thank the reviewer for this comment. We have revised the recommendation section based on this recommendation.

**Reviewer #4:**

**Comment: Many thanks for the chance to review this manuscript. I think the findings of this review have the potential of improving the healthcare system in a populated country like India, and possibly advance digital health adoption and research. However, I think the manuscript currently have some major flaws, particularly on the methods. The methods need to be more thorough and explicit that would allow for a replication. The use of a single database for a scoping review is a HUGE flaw that cannot possibly be compensated for the lack of resources. If this is a scoping review, then the authors should consider including other materials (unless they did not find any) beyond published peer-reviewed publications.**

Response: We agree with the reviewer and have acknowledged this as a major limitation. That said, we would like to provide some context for our choice. As described in the review protocol attached in the supplement, this review is part of a larger set of reviews synthesized to inform the ongoing work of the Lancet Commission for Reimagining India’s Health System. The choice for Pubmed was based on the fact that it is an open-access, specialized, and curated database. We wanted to consider literature that passed the peer review check as the minimum requirement for scientific credibility and integrity to use them to inform the Commission confidently. Hence, sources of gray literature, such as Google Scholar, were avoided. Also, we wanted to ensure that the search is replicable for free around the world (open access). Hence, cost-prohibitive sources such as Web of Science and Scopus were avoided.

**Comment: The second paragraph in the introduction is not logically connected with the third one. The authors should consider making a logical connection or better still, remove it as it’s a bit off track.**

Response: We thank the reviewer for the comment. We have added a line at the end of the second paragraph to improve the flow of the content.

**Comment: The methods is not robust enough: the authors should provide more information on the search strategy. What search terms were used and for which databases (authors only mentioned PubMed). If the search strategy was based on a framework, the authors should provide a background to the WHO Action framework , the authors provide a background to that framework and explain how the framework was used in this review. As it is now, it is unclear how the authors conducted their search.**

Response: We thank the reviewer for their comment. The search strategy is mentioned in the S1 panel, which is part of the supplementary material, the healthcare worker cadres are mentioned in S2 panel and the WHO action framework is mentioned in S1 Table. We have also explained why we used the WHO action framework for HRH in the methodology section.

**Comment: The use of only one database is hugely problematic and its not something that can simply be acknowledged as a limitation. By using only PubMed, my believe is that a significant number of papers that meet the inclusion criteria but were not indexed in PubMed might have been excluded.**

Response: We agree with the reviewer and have acknowledged this as a major limitation. However, we cannot comment on whether the number of studies left out is “significant” or not.

**Comment: The authors removed some 203 duplicates, even though they claimed to have used only PubMed and I am wondering how that happened. Is it theoretically impossible for a paper to be indexed twice in the same database. You only remove duplicates when you search multiple sources and then remove papers that might have been indexed across two or more databases. So if a single database was used, how come duplicates were removed ?**

Response: We thank the reviewer for this comment. Our final search was a combination of articles obtained from two search strategies as highlighted in the supplementary file (S1 Panel). We have edited the S1 Panel to add more clarity.

**Comment: I am not too sure if this is a scoping review or a systematic review. If this is a scoping review, then the authors should include other materials (published or unpublished) beyond peer-reviewed research papers. As it stands now, the authors excluded materials that ordinarily would qualify for inclusion in a scoping review.**

Response: We thank the reviewer for this comment. Based on the reviewer’s suggestion, we have now changed to note it as a systematic review.

**Comment: The authors stated in the methods overview that the study adopted Arksey and O’Malley methodological framework. However, the methods outlined is a bit inconsistent with Arksey and O’Malley framework.**

Response: We thank the reviewer for this comment. Since based on the reviewer’s previous comment we are mentioning this to be a systematic review, we have removed Arksey O’Malley framework from our methodology as it is meant for scoping reviews.

**Comment: The screening processes for arriving at the final papers as shown on the Prisma Diagram appears problematic. At the screening stage, the authors removed 439 papers due to inappropriate study design. I am wondering how they determined the study design when they were screening titles and abstracts because you can only determine the appropriateness of a study design until you review the full text. The authors also removed papers at the screening stage based on other criteria that can only be determined in full text review.**

Response: The abstracts briefly mention the methodology, which most often mentions the study design. Similarly, the authors were able to exclude studies by identifying other characteristics of the study by going through the abstract. Details of study design inclusion and excursion criteria are mentioned in the methods section and also are provided in the protocol registered in OSF [https://doi.org/10.17605/OSF.IO/KQ3U9].

**Comment: The results is a bit scanty: More information is needed to shed light on the barriers and facilitators**

Response: We thank the reviewer for the comment. However, it would be helpful if the reviewer could provide some specific feedback where they felt more elaboration or explanation is needed. Currently, the results section has three tables and ten figures. These and the accompanying text provide a decent summary of the principal findings. However, if there are subsections that the reviewer thinks need more clarity, we would be happy to work on those based on any specific feedback.
